# In-vitro Immunomodulatory activity of *Azadirachta indica* A.Juss. Ethanol: water mixture against HIV associated chronic CD4^+^ T-cell activation/ exhaustion

**DOI:** 10.1186/s12906-021-03288-0

**Published:** 2021-04-09

**Authors:** Omalla A. Olwenyi, Bannet Asingura, Prossy Naluyima, Godwin Upoki Anywar, Justine Nalunga, Mariam Nakabuye, Michael Semwogerere, Bernard Bagaya, Fatim Cham, Allan Tindikahwa, Francis Kiweewa, Eliezer Z. Lichter, Anthony T. Podany, Courtney V. Fletcher, Siddappa N. Byrareddy, Hannah Kibuuka

**Affiliations:** 1 Makerere University, Walter Reed Project, P.O Box 16524, Kampala, Uganda; 2 Department of Pathology and Microbiology, University of Nebraska Medical Center, Omaha, NE USA; 3 Department of Pharmacology and Experimental Neuroscience, University of Nebraska Medical Center, Omaha, NE USA; 4 Department of Immunology and Molecular Biology, College of Health Sciences, Makerere University, Kampala, Uganda; 5 Department of Plant Sciences, Microbiology & Biotechnology, College of Natural Sciences, Makerere University, Kampala, Uganda; 6 Department of Biochemistry and Molecular Biology, College of Medicine, University of Nebraska Medical Center, Omaha, NE USA; 7 Antiviral Pharmacology Laboratory, University of Nebraska Medical Center (UNMC) Center for Drug Discovery, Omaha, NE USA

**Keywords:** Immunomodulation, CD4^+^ T cell activation/exhaustion, *Azadirachta indica (A. indica)* ethanol: water mixture, Staphylococcal enterotoxin B (SEB), Microbial translocation

## Abstract

**Background:**

In Sub-Saharan Africa, herbal therapy continues to be utilized for HIV-1 disease management. However, the therapeutic benefits of these substances remain ambiguous. To date, little is known about the effects of these plant extracts on chronic CD4 + T-cell activation and exhaustion which is partly driven by HIV-1 associated microbial translocation.

**Methods:**

Effects of *Azadirachta indica, Momordica foetida and Moringa oleifera* ethanol: water mixtures on cell viability were evaluated using the Guava PCA system. Then, an in-vitro cell culture model was developed to mimic CD4+ T cell exposures to antigens following HIV-1 microbial translocation. In this, peripheral blood mononuclear cells (PBMCs) isolated from HIV negative (*n* = 13), viral load < 1000 copies per mL (*n* = 10) and viral load > 1000 copies per mL (*n* = 6) study participants from rural Uganda were treated with Staphylococcus enterotoxin B (SEB). Then, the candidate plant extract (*A. indica*) was added to test the potential to inhibit corresponding CD4+ T cell activation. Following BD Facs Canto II event acquisition, variations in %CD38, %CD69, Human Leukocyte Antigen -DR (HLA-DR), Programmed cell death protein 1 (PD-1), T-cell immunoglobulin and mucin domain-containing protein 3 (Tim-3), interferon gamma (IFN γ) and interleukin 2 (IL-2) CD4 + T cell expression were evaluated.

**Results:**

Following exposure to SEB, only *A. indica* demonstrated a concentration-dependent ability to downregulate the levels of CD4 + T cell activation. At the final concentration of 0.500 μg/mL of *A. indica,* a significant downregulation of CD4 + CD38 + HLA-DR+ expression was observed in HIV negative (*p* < 0.0001) and both HIV infected groups (*P* = 0.0313). This plant extract also significantly lowered SEB induced % CD4+ T cell HLADR, PD-1 and Tim-3 levels. PD-1 and CD69 markers were only significantly downmodulated in only the HIV negative ((*p* = 0.0001 and *p* = 0.0078 respectively) and viral load< 1000 copies per ml (p = 0.0078) groups.

**Conclusion:**

*A. indica* exhibited the in-vitro immunomodulatory potential to inhibit the continuum of SEB induced CD4+ T-cell activation/ exhaustion without impacting general T-cell specific functions such as cytokine secretion. Additional studies are needed to confirm *A. indica* as a source of natural products for targeting persistent immune activation and inflammation during ART.

**Supplementary Information:**

The online version contains supplementary material available at 10.1186/s12906-021-03288-0.

## Background

Life prolonging antiretroviral therapy (ART) has remarkably improved the quality of life of people living with HIV (PLWH) worldwide. However, in resource-limited settings such as sub-Saharan Africa (SSA), incapacitated health care systems limit access to ART [1–3]. In these settings, cultural differences and poor health care force PLWH resort to herbal therapy for alternative/ complementary treatment [4–7]. Hence, it is common for PLWH who are actively enrolled on ART to concurrently use traditional herbal medicines [8]. This warrants the need for studies investigating any immune-boosting benefits arising from the routine use of medicinal plants for HIV diseases management.

HIV principally infects CD4^+^ T cells and skews diverse cellular pathways to favour its replication [9]. This is also accompanied by persistent CD4^+^ T cell activation as denoted by increased surface expression of (1) the early activation marker CD69, (2) the antigen presenting molecule Human Leukocyte Antigen – DR (HLA-DR), (3) the metabolite marker CD38 [10, 11]. This state of chronic immune activation is majorly driven by continuous responses to bacterial antigens arising from gut microbial translocation [12].

Early in infection, HIV causes irreparable damage to the gut epithelium. This causes gut microbes and bacterial products to transverse the gut protective barrier [13]. This sets the stage for the establishment of systemic immune activation and subsequent HIV pathogenesis. As such, chronic T cell activation has been linked to accelerated progression to AIDS and is suggested as an even better predictor of AIDS events compared to viral load and CD4^+^ T cell count in HIV infected individuals [14]. In addition, the continuous response of CD4^+^ T cells towards various antigenic stimuli during the course of HIV infection leads to increased CD4^+^ T cell exhaustion. This is exemplified by the elevated surface expression of program cell death protein 1 (PD-1) and Tim 3 checkpoint inhibitory receptors [15]. In summary, the increased expression of multiple exhaustion markers on CD4^+^ T cells is associated with the loss of HIV-specific cell function. This is characterized by a reduction in Interleukin 2 (IL-2) and Tumour Necrosis Factor alpha (TNFα) cytokine secretion [16, 17].

Blockade of exhaustion markers like anti PD-1 has previously been shown to improve disease outcomes by limiting hyper immune activation and promoting CD4^+^ T cell function. This later facilitates improved immunological control of the virus [18–20]. Succinctly, Mylvaganam GH et al, suggest that the administration of therapeutics designed to limit T cell activation / exhaustion could provide additional benefits when combined with ART [19]. In SSA, HIV-1 infected individuals have higher levels of T cell activation compared to persons from developed countries. This is mainly due to the high burden of endemic pathogens that continuously activate the immune system [21–23]. This further emphasizes the need for urgent therapy-based modalities focused on dampening hyper-immune activation within these communities.

As recently as 2019, Bourke et al. showed that long-term use of the antibiotic Cotrimoxazole together with ART lowers monocyte secretion of pro-inflammatory cytokines. However, Cotrimoxazole did not reduce the levels of immune activation in CD4^+^ T cells following in-vitro stimulation with the bacterial bi-product Staphylococcal Enterotoxin B (SEB) [23].

To advance the search for compounds targeting HIV-1 associated CD4^+^ T-cell activation, we tested ethanolic plant extracts for their potential immune-modulatory potential. Based on a library of medicinal plants reportedly used by PLWH in SSA, the following plant species were selected: *Momordica foetida* Schumach, *Azadirachta indicia* A. Juss. and *Moringa oleifera* [7, 24, 25].

Here, we report that following in-vitro stimulation of HIV infected peripheral blood mononuclear cells (PBMCs) with SEB, *A. indica* leaf ethanol: water mixture is capable of down-regulating CD4+ T-cell activation and exhaustion. This suggests that *A. indica* could serve as a potential source of bioactive compounds that could be developed for targeting chronic immune activation in PLWH.

## Methods

### Preparation of plant extracts

#### Plant collection and preparation

For the collection of plant specimens, permission was sought from Uganda National Council for Science and Technology (UNCST). Later on, guidance was obtained from the Department of Plant Sciences, Microbiology & Biotechnology at Makerere University. Fresh *M. foetida* (leaves)*, A. indica* (leaves) and *M. oleifera* (leaves) were harvested from Mabira Central Forest Reserve in Uganda. The plant specimens were identified by a botanist (Godwin Anywar, Department of Plant Sciences, Microbiology and Biotechnology Makerere University) using field guides [26, 27]. Voucher specimens of the selected species were collected following standard procedures described in Martin et al 1995 [28] and deposited at the Makerere University herbarium for confirmation. All plant materials were processed following the World Health Organisation (WHO) guidelines on Good Agricultural and Collection Practices (GACP) for medicinal plants (WHO, 2003) [29].

#### Plant extraction

The harvested plant parts were then washed separately with distilled water to remove debris, oven-dried at 40 °C for 5 days and ground to a fine powder using a blender. Whilst processing the plant species, the blender was thoroughly cleaned using 70% ethanol. Solid-liquid extraction was performed by soaking 200 g of fine powder from each plant specimen in 400 mL of 70% ethanol for 24 h. The resultant crude extracts were separately filtered through Whatman Grade 1 qualitative filter paper (Sigma-Aldrich, USA) and precipitated using a rotary evaporator (Buchi® rotary evaporator Model, R-205) at reduced pressure. Then the precipitates were dried overnight in a desiccator. The final yields of the dried crude ethanol-water mixtures ranged from 20 to 25%. Following this, 0.98 g of *A. indicia,* 0.24 g of *M. foetida*, and 1.07 g of *M. oleifera* dissolved in 1 ml of dimethyl sulfoxide (DMSO) (Sigma-Aldrich, USA) to make respective stock solutions. The stock solutions were later filtered through 0.22 μm Millex-GP syringe filters (Merck, Darmstadt Germany). The final plant ethanol-water mixtures were stored at 4 °C and later reconstituted for further experimentation.

#### Study participants and consent information

All study procedures were approved by the Makerere University College of Health Sciences School of Public Health Higher Degrees, Research and Ethics Committee (HDREC), UNCST. For in-vitro screening of the immunomodulatory effects of the selected plant extracts, HIV-1 infected individuals on ART and healthy HIV negative individuals residing in Kayunga district, in central Uganda were targeted for enrolment. Study participants were eligible for enrolment if they: (1) provided written informed consent. (2) were aged between 18 and 49 years. (3) had 6-month medical data including at least a complete blood count (CBC) profile, either viral load (VL) result and/ or CD4 count (if HIV infected). (4) had normal blood haemoglobin count. (5) had not reported herbal medicine use within 7 days of blood sample collection. A questionnaire was administered to capture: the frequency of herbal medicine use, commonly used herbal plants, method of herbal extract preparation and whether several plant varieties were used concurrently (Supplementary document).

#### HIV screening and viral load testing

In all study participants, HIV-1 infection screening was performed sequentially using Determine™ HIV-1/2 AG/AB COMBO (Abbott Laboratories, Illinois, USA), Chembio HIV 1/2 STAT-PAK® Assay (Chembio Diagnostics, New York, USA) and Uni-Gold Recombigen® HIV-1/2 (Trinity Biotech, Wicklow, Ireland) following respective manufacturers’ instructions [30–32]. Thereafter, in HIV positive study participants, HIV-1 viral load (VL) was determined using the COBAS® AmpliPrep/COBAS® TaqMan® HIV-1 Test, v2.0 (Roche Holding AG, Basel, Switzerland) [33].

#### Blood sample collection and PBMC processing

Sterile whole blood samples were collected from consenting study participants in 8.5 ml BD vacutainer acid citrate dextrose (ACD) tubes [34] and PBMCs were isolated within 8 h of sample collection using the Ficoll-Hypaque density centrifugation technique as previously described [35]. PBMC counts and viability were determined by Muse® Count & Viability Assay kit (Merck, Darmstadt Germany), a Guava Personal Cell Analysis (PCA) system, and Guava Viacount CytoSoft™ Software Version 6.2.2 (Guava Technologies, USA) following manufacturer’s instructions [36]. PBMCs with cell viability greater than 85% were cryopreserved at 1 X 10^7^ cells/mL in freeze media containing 10% DMSO, 10% heat inactivated fetal bovine serum (FBS), 1% penicillin-streptomycin (pen-strep), 2% HEPES buffer, 2% L-glutamine and 75% RPMI-1640. All reagents used in the freeze media were obtained from St. Louis, MO Sigma-Aldrich, USA.

#### Cytotoxicity screening based on cell viability testing

To evaluate the cytotoxicity of the plant extracts, cryopreserved PBMCs from HIV negative blood packs were thawed and rested overnight in 10% complete media (10% FBS, 1% pen-strep, 2% HEPES buffer, 2% L-glutamine and 85% RPMI-1640) at 37 °C, 5% CO_2_ and 90% relative humidity (RH). All reagents in 10% complete media were obtained from St. Louis, MO Sigma-Aldrich, USA. Then PBMC counts and viability assessments were performed as previously described [36, 37] and PBMCs with greater than 85% cell viability were seeded in polystyrene 96-well plates (1 X 10^5^ cells/well) containing 200 μL of 10% complete media per well with varying concentrations (serial dilutions) of either SEB (St. Louis, MO Sigma-Aldrich, USA) or cyclosporine A (St. Louis, MO Sigma-Aldrich, USA) or the plant extracts. The PBMCs were then incubated at 37 °C, 5% CO_2_ and 90% RH for 36 h with regular replenishment (12-h intervals) of the culture media. Finally, PBMC counts and viability assessments were performed as previously described [36, 37] to determine the maximum concentration of each plant extract at which the lowest level of cell death is observed. Only the maximum concentration of each ethanol-water mixture at which PBMC viability was maintained above 80% was considered for further study.

### Evaluating the effect of plant ethanol-water mixtures on CD4^+^ T-cell activation and exhaustion following SEB stimulation

#### In-vitro cell culture and stimulation

Following SEB stimulation, cryopreserved PBMCs from HIV negative blood packs (*n* = 5) were seeded in a polystyrene 96-well plate (1 X 10^6^ cells/well) containing 200 μL of 10% complete media per well with different treatment conditions. These included: 1 μg/mL SEB (positive control); 0.015 μg/mL of cyclosporine A (negative control) [38]; 1 μg/mL SEB and 0.015 μg/mL cyclosporin A (Cyclo + SEB); 0.5 μg/mL *A. indica* and 1 μg/mL SEB (*A. indica* + SEB). In addition, 0.5 μg/mL *M. oleifera* and 1 μg/mL SEB (*M. oleifera* + SEB), 0.5 μg/ml *M. foetida* and 1 μg/mL SEB (*M. foetida* + SEB); and finally, 0.5 μg/mL of *A. indica* only were also included. An unstimulated condition was also included as a negative control and incubation performed at 37 °C, 5% CO_2_ and 90% RH for 24 h.

#### Flow cytometry surface staining

The treated PBMCs were then washed twice in phosphate buffer saline (PBS) and stained with BD Multitest™ CD4 (clone SK3) FITC/CD38 (clone HB7) PE/CD3 (clone SK7) PerCP/Anti-HLA-DR (clone L243) APC (BD Biosciences, San Jose, CA), Tim-3 BV421 (Clone 7D3, BD Biosciences, San Jose CA), PD-1 PE-Cy7 (Clone EH12.1, BD Biosciences, San Jose CA), CD69 APC-H7 (Clone FN50, BD Biosciences, San Jose, CA) and LIVE/DEAD™ Fixable Aqua Stain (Invitrogen, Carlsbad, CA). The PBMCs were then fixed with 2% paraformaldehyde (PFA), washed again in PBS and events acquired using a BD FACS Canto II. Fluorescence minus one (FMO) controls were used to set cut-off gates for markers that did not have a clear resolution during data analysis (Supplementary Figure 1).

#### Testing the potential of the a.indica ethanol-water mixture to downregulate CD4+ T cell activation/ exhaustion in HIV infected individuals

After observing that only the *A. indica* ethanol-water mixture reduced SEB-induced CD4^+^ T cell activation, HIV negative PBMCs (1 X 10^6^ cells/well) were treated with increasing concentrations of *A. indica* extracts (0.0 to 0.5 μg/mL) and 1 μg/mL of SEB in 10% complete media for 24-h at 37 °C, 5% CO_2_ and 90% RH. Later experiments were then performed with a focused intent of testing the ability of *A. indica* to reduce the levels of chronic CD4^+^ T cell activation/ exhaustion in HIV negative (*n* = 13), viral load less than 1000 copies per ml (*n* = 10) and viral load greater than 1000 copies per ml (*n* = 6) study groups.

#### Evaluating the effect of *A. indica* extract on gag-specific CD4^+^ T-cell cytokine responses

After observing that the *A. indica* extract downmodulated SEB-induced CD4^+^ T cell activation and exhaustion, next we sought to determine whether this was accompanied by altered HIV specific CD4^+^ T cell IL2 and INF-γ responses. Cryopreserved PBMCs were thawed and rested overnight at 37 °C, 5% CO_2_ and 90% RH. Then the PBMCs were seeded in a polystyrene 96-well plate (1 X 10^6^ cells/well) containing 200 μL of 10% complete media per well with either of the following treatment conditions: 2.5 μg/mL of Gag i.e. PepMix™ HIV GAG (Ultra, JPT peptides Inc., Berlin, Germany) [39], 0.5 μg/mL *A. indica* and 2.5 μg/mL Gag (*A. indica* + Gag), 2.5 μg/mL Gag and 2 μg/mL SEB (positive control). Afterwards, 1 μg/mL of anti-CD28 (Clone CD28.2, BD Biosciences, San Jose CA) and 1 μg/mL of anti-CD49 (Clone L25, BD Biosciences, San Jose CA) were added as co-stimulants, then 2 μg/mL brefeldin A (BD Biosciences, San Jose CA) and 1 μM monensin (BD Biosciences, San Jose CA) were added to block cytokine efflux from the Golgi apparatus. The 96-well plates were then incubated at 37 °C, 5% CO_2_ and 90% relative humidity (RH) for 10 h. After, the PBMCs were stained with LIVE/DEAD™ Fixable Aqua Stain (Invitrogen, Carlsbad CA), washed in PBS and then Fc receptor blocked using Human BD Fc Block™ (BD Biosciences, San Jose, CA). Cell surface staining was then performed using CD4 PE Cy7 (Clone SK3 BD Biosciences, San Jose, CA), CD8 PE Cy5 (Clone RPA-T8 BD Biosciences, San Jose, CA) and CD3 APC-H7 (Clone SK7, BD Biosciences, San Jose, CA). Following this, the PBMCs were fixed using 2% PFA and permeabilized with BD permeabilization buffer prior to intracellular staining with IL-2 BV421 (Clone MQ1-17H12, BD Biosciences, San Jose, CA) and interferon gamma (IFN-γ) APC (Clone B27, BD Pharmingen™, San Jose, CA). Finally, events were acquired within 24 h using a BD FACSCanto™ II. Corresponding Flow Cytometry Standard (FCS) data files were evaluated following the gating strategies in Supplementary Figures 1 and 2.

### Statistical analysis

FCS files were analysed using FlowJo Version 10.0.7 [40] and all statistical analyses were performed using GraphPad Prism version 7.0 software [41]. Within each group, paired data analyses were calculated using the Wilcoxon matched-pairs signed rank test for differences within medians. Results with *p*-values < 0.05 were considered statistically significant.

## Results

### Study participant characteristics

Between June and August 2017, 29 study participants aged between 18 and 49 years were enrolled in the study. All participants had normal complete blood counts (CBC) /blood haemoglobin counts. Their median weight was 62 (50.0–81.3) kgs. Of the enrolled participants, 15 (52%) were female while 14 (48%) were male. Notably, 45% of the study participants recruited were HIV-1 positive. Of the 16 HIV-1 infected subjects, 9 were females and 7 were males. Within the HIV-1 infected participants, 9 had VL less than 1000 copies/mL and were considered virally ‘suppressed’ according to recommended guidelines for VL monitoring in resource-limited settings [42, 43]. Lastly, of all the recruited study participants, 9 (31%) reported herbal use. Amongst these herbal users, 7 were female while 2 were male. Our self-reporting questionnaire revealed that 4 of the 9 herbal users prepared the herbal concoctions themselves (self-preparation), (Table 1).

**Table 1:** Demographics of Study Participants

**Age**	
Study participants	29
Over 18 years	29
**Immune Status**	
Normal complete blood count (Hemoglobin > 12 g/dl)	29
Weight > 50 Kg	29
Weight, Kg (range)	62 (50–81.3)
**Sex**	
Male (%)	14 (48)
Female (%)	15 (52)
**HIV Infection Status**	
Negative	13
Positive (VL < 1000 copies/ml)	9
Negative (VL > 1000 copies/ml)	7
**Documented Herbal Medicine Users**	
*Vernonia amygdalina*	4
*Momordica foetida*	3
*Bidens pilosa*	1
*Hoslundia opposita*	1
**Frequency of Herbal Medicine Use**	
History of herbal use	9
At least once per year	7
Monthly herbal medicine	1
Prepared the herbal extracts themselves (self-preparation)	4
Used a combination of at least 2 plants	4
**Tobacco smokers**	**1**
**Alcohol consumers**	**4**

From: In-Vitro Screening of Selected Tropical African Plants reveals the Potential Immunomodulatory Activity of *Azadirachta indica* Ethanolic Extract against HIV Associated Chronic CD4^+^ T-Cell Activation/ Exhaustion

### Effect of plant extracts on PBMC viability

The cytotoxicity experiments were conducted for 24 h to test the ability of the extracts to maintain plasma membrane integrity and minimize cell death. Also, an 80% cell viability threshold was selected based on previous drug screening studies which showed that drug concentrations which maintain cell viabilities greater than 80% in culture usually possess acceptable toxicity profiles [44, 45].

**Fig. 1:**
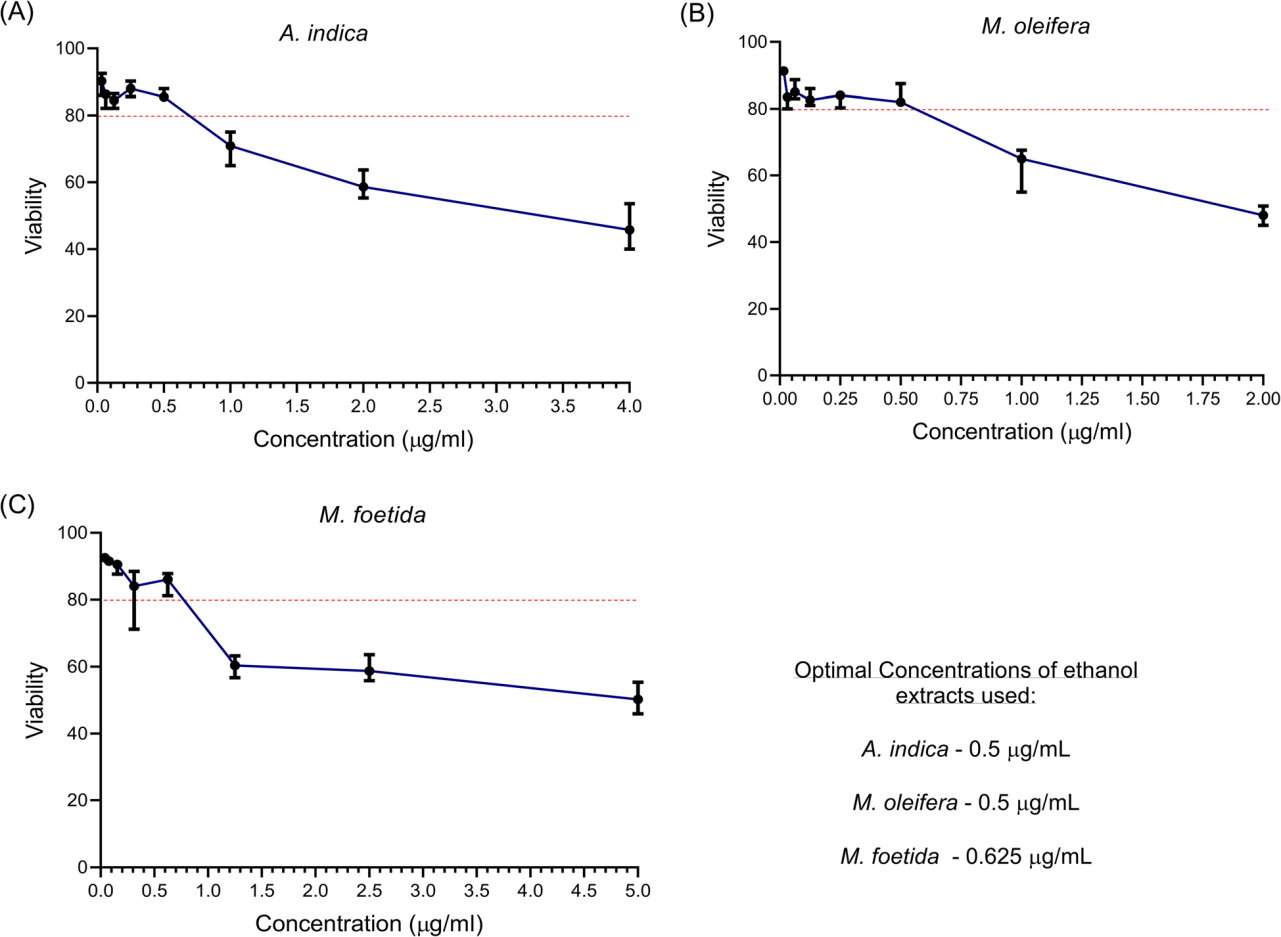
Evaluation of the effects of various concentrations of different plant extracts on cell viabilities of thawed PBMCs collected from three independent study participants. Cytotoxic effects of (**a**) *A. indica* (**b**) *M. oleifera* (**c**) *M. foetida* in thawed PBMCs following incubation for 24 h. Optimal concentrations were evaluated by determining the highest concentration at which a viability greater than 80% was obtained for each plant extract

After 24 h of PBMC culture, a general concentration-dependent trend was observed whereby increasing concentrations of plant extracts was accompanied by a simultaneous decline in PBMC viability (Fig. 1). The *A. indica* extract exhibited a cytotoxicity profile that was characterized by fluctuating PBMC viability at concentrations below 0.5 μg/mL that was maintained above the cut-off of 80% viability. Above 0.5 μg/mL, there was a steady decline in viability (Fig. 1a). Conversely for the *M. oleifera* extract, above 0.5 μg/mL, a progressive loss in cell viability that dropped below the 80% threshold was noticed (Fig. 1b). In turn, for the *M. foetida* extract, PBMC viability fluctuated between a range of 70 to 90% at concentrations between 0.0 to 0.5 μg/mL. This was followed by a steady decline in viability, (less than 80%), at concentrations above 0.5 μg/mL (Fig. 1c). Viability was maintained following prolonged cellular exposures to *A. indica* that lasted up to 48 h.

### Effect of plant extracts on CD4^+^ T-cell activation following in-vitro stimulation with SEB

**Fig. 2:**
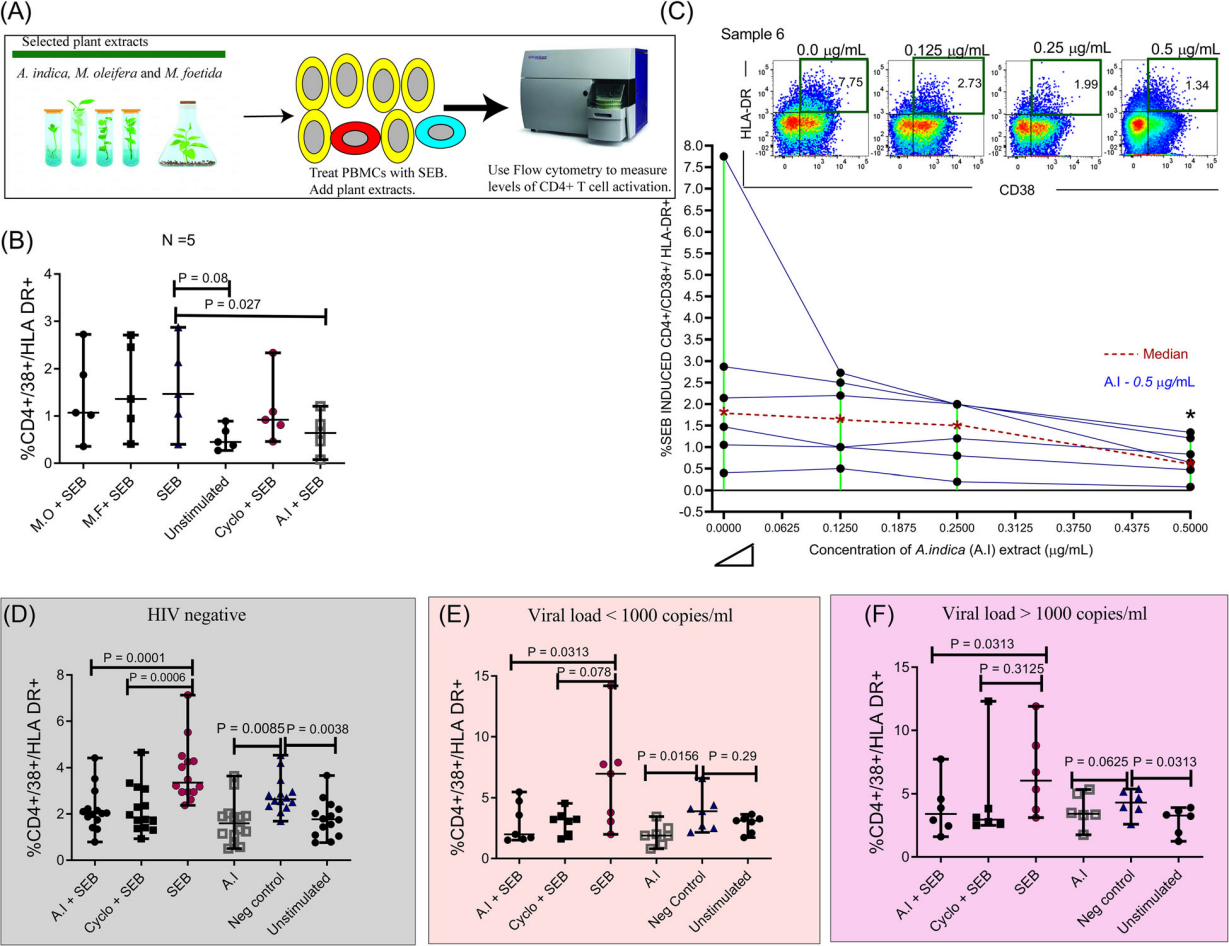
*A. indica* down-modulates the levels of %CD4 + CD38 + HLA-DR+ cells following treatment with SEB. **a** Experimental schema showing plant extracts (*A. indica*, *M. oleifera* and *M. foetida*) that were selected for further testing of their ability to reduce the levels of CD4+ T cell activation following exposure to bacterial antigens (SEB). **b** Levels of %CD4 + CD38 + HLA-DR+ levels following treatment with SEB alone and the various plant extracts (*A. indica* (A.I), *M. oleifera* (M.O) and *M. foetida* (M.F)). **c** Dose responses of A. I (0.0 μg/ mL, 0.125 μg/ mL, 0.250 μg/ mL and 0.50 μg/ mL) on SEB induced %CD4 + CD38 + HLA-DR+ levels. Levels of SEB induced %CD4 + CD38 + HLA-DR+ cells after addition of A. I + SEB, Cyclosporine (Cyclo) + SEB, SEB, A. I negative (Neg) control (Cyclo alone) and unstimulated conditions in **d** HIV negative **e** viral load < 1000 copies/ mL and **f** viral load > 1000 copies/ mL study groups. * shows *p* < 0.05 and ** represents *p* < 0.01 paired significant difference across studied groups. Statistical analysis was performed using the Wilcoxon matched-pairs signed rank test. Each datapoint represents results obtained from individual donor samples

*A. indica*, *M. foetida* and *M. oleifera* extracts were further evaluated to determine their effect on CD4^+^ T cell activation (%CD4 + CD38 + HLA-DR+) following stimulation with SEB. Preliminary assessment using PBMCs from five HIV-1 negative individuals showed that only the *A. indica extract* reduced SEB-induced CD4^+^ T-cell activation since the %CD4 + CD38 + HLA-DR+ cells reduced from 1.470 (0.400–2.870) % in the positive control to 0.640 (0.079–1.210) % in the *A. indica +* SEB treatment condition, (*p =* 0.027) (Fig. 2b). In addition, the observed downregulation of CD4^+^ T cell activation was concentration-dependent at a range of 0.0 to 0.5 μg/mL since increasing concentrations of the *A. indica* extract decreased the level of SEB-induced co-expression of CD38 and HLA-DR on CD4^+^ T cells (Fig. 2c).

Based on the above results, *A. indica* was selected as opposed to the other plant extracts tested in this study. This was because that it was the only candidate extract that significantly downregulated CD38+/HLA-DR+ co-expression in SEB stimulated CD4+ T cells in a concentration-dependent manner. Thus, *A. indica* was tested on PBMCs from study participants with known HIV-1 status, CBC and haemoglobin count. In HIV negative study participants, 0.5 μg/mL of *A. indica* reduced SEB-induced CD4^+^ T cell activation from 3.355 (2.380–7.130) % in the positive control to 2.030 (0.79–4.420) %CD4 + CD38 + HLA-DR+ cells (*p* = 0.0001) in the *A. indica +* SEB treatment condition. In parallel comparative experiments, the immunomodulatory effects of *A. indica* extract mirrored the known inhibitory properties of cyclosporin A which also reduced SEB-induced CD4^+^ T cell activation from 3.355 (2.380–7.130) % to 1.860 (0.9300–4.660) % CD4 + CD38 + HLA-DR+ cells (*p* = 0.0006) (Fig. 2d). In separate conditions where the PBMCs obtained from HIV-1 negative individuals were not exposed to SEB, distinct changes in % CD4 + CD38 + HLA-DR+ cells were seen. Notably, the 0.5 μg/mL *A. indica* alone condition had similar %CD4 + CD38 + HLA-DR+ frequencies as the unstimulated condition. However, the *0.5* μg/mL *A. indica* alone (1.595 (0.500–3.640)) % and the unstimulated condition 1.770 (0.760–3.660) % possessed lower levels of % CD4 + CD38 + HLA-DR+ cells than the 0.015 μg/mL of cyclosporine A alone/ negative control condition (2.635 (1.690–4.540) %), (*p* = 0.0085 and *p* = 0.0038 respectively) (Fig. 2d). Similar inhibitory effects were observed in HIV-1 infected participants. The *A. indica* extract reduced SEB-induced CD4^+^ T cell activation in PBMCs by greater than 1.5 folds from participants with or without suppressed viremia, (*p* = 0.0313 in both cases) (Fig. 2e and f). Likewise, as observed in the HIV-1 negative participants, the *A. indica* alone condition had close to two-fold lower levels of % CD4 + CD38 + HLA-DR+ cells in comparison to cyclosporine A alone conditions in PBMCs from participants with suppressed viremia (*p* = 0.0156) (Fig. 2e).

### Effect of the *A. indica* ethanol-water mixture on early CD4^+^ T cell activation and subsequent exhaustion

**Fig. 3:**
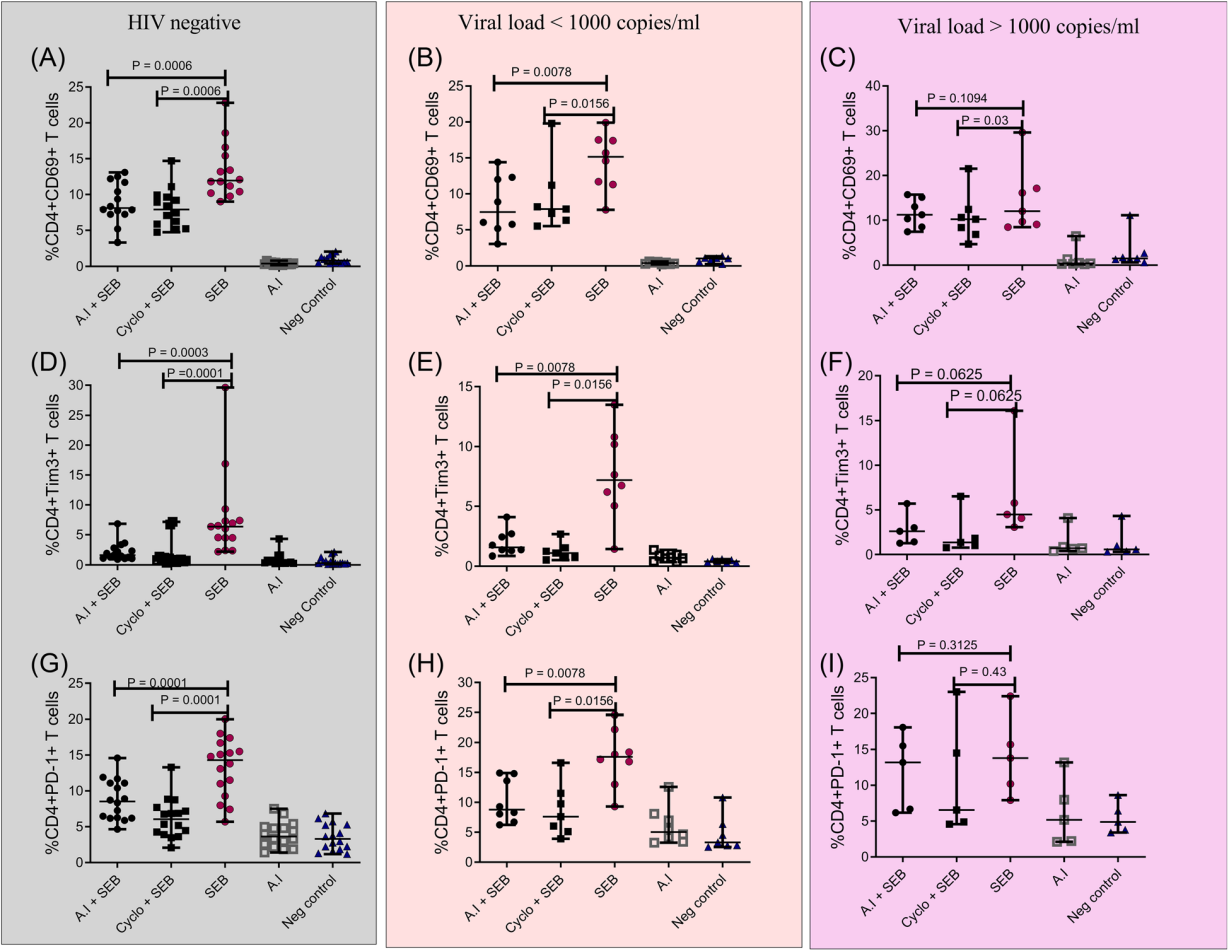
*A. indica* also reduces the levels of other markers of SEB induced immune activation (CD69) and exhaustion (PD-1 and Tim-3) in PBMCs obtained from study participants. Levels of SEB induced %CD4+/CD69+ T cells after addition of *A. indica* (A.I) + SEB, Cyclosporine (Cyclo) + SEB, SEB, A. I negative (Neg) control (Cyclo alone) and unstimulated conditions in (**a**) HIV negative (**b**) viral load < 1000 copies/ mL and (**c**) viral load > 1000 copies/ mL study groups. %CD4+/Tim3+ T cell levels following the maintenance of similar stimulatory conditions as in (**a**-**c**) that involved addition of A. I + SEB, Cyclo + SEB, SEB, A. I*,* negative (Neg) control (Cyclo alone) and the unstimulated states in (**d**) HIV negative (**e**) viral load < 1000 copies/ mL and (**f**) viral load > 1000 copies/ mL study groups. Percent levels of CD4+/PD-1+ T cells following stimulation with A. I + SEB, Cyclo + SEB, SEB, A. I, negative (Neg) control (Cyclo alone) and the unstimulated states in (**a**) HIV negative (**b**) viral load < 1000 copies/ mL and (**c**) viral load > 1000 copies/ mL study groups. Statistical evaluation was carried out using the Wilcoxon matched-pairs signed rank test. Each datapoint represents results obtained from individual donor samples

Having noticed that *A.indica* down modulated levels of CD4 + CD38 + HLA-DR+ co-expression, we sought to test whether this plant extract would have effects on different stages of CD4+ T cell activation. The *A. indica extract* reduced the expression of the early T-cell activation marker CD69 and PD-1/ Tim-3 exhaustion markers in a similar fashion as cyclosporine A. In HIV negative study participants, *A. indica* extract reduced SEB-induced CD69 expression from 11.95 (9.00–22.80) % in the positive control to 8.12 (3.35–13.10) % in the *A. indica +* SEB treatment condition (*p* = 0.0006). In parallel, the observed *A. indica* extract mediated reduction of SEB-induced CD69 expression was equivalent to the anticipated inhibitory effect of cyclosporine A (p = 0.0006) (Fig. 3a). A similar trend was observed in participants with suppressed viremia where both the *A. indica* extract and cyclosporine A reduced SEB-induced CD69 expression by at least 1.4 folds (*p* = 0.0078) and (p = 0.0156) respectively (Fig. 3b). However, *A. indica’*s inhibitory effect on CD69 expression was not replicated in CD4^+^ T cells from participants with VL greater than 1000 copies/mL (*p* = 0.11). Nonetheless, cyclosporine A maintained CD69 inhibition (*p* = 0.03) within this group (Fig. 3c). With regards to CD4^+^ T cell exhaustion, again *A. indica* extract mirrored cyclosporine A-induced inhibition by lowering Tim-3 (*p* = 0.0003) and PD-1 (*p* = 0.0001) in CD4^+^ T cells from HIV negative (Fig. 3d and Fig. 3g) and suppressed VL participants (p = 0.0078 for both markers) by 1.7 to 4.2-fold decrements (Fig. 3e and Fig. 3h). However, both the *A. indica* extract and cyclosporine A did not reduce SEB-induced Tim-3 and PD-1 in CD4^+^ T cells from participants with VL greater than 1000 copies/mL (Fig. 3f and i).

### Effect of the *A. indica* ethanol-water mixture on gag-specific CD4^+^ T cell IFN-γ and IL-2 response

**Fig. 4:**
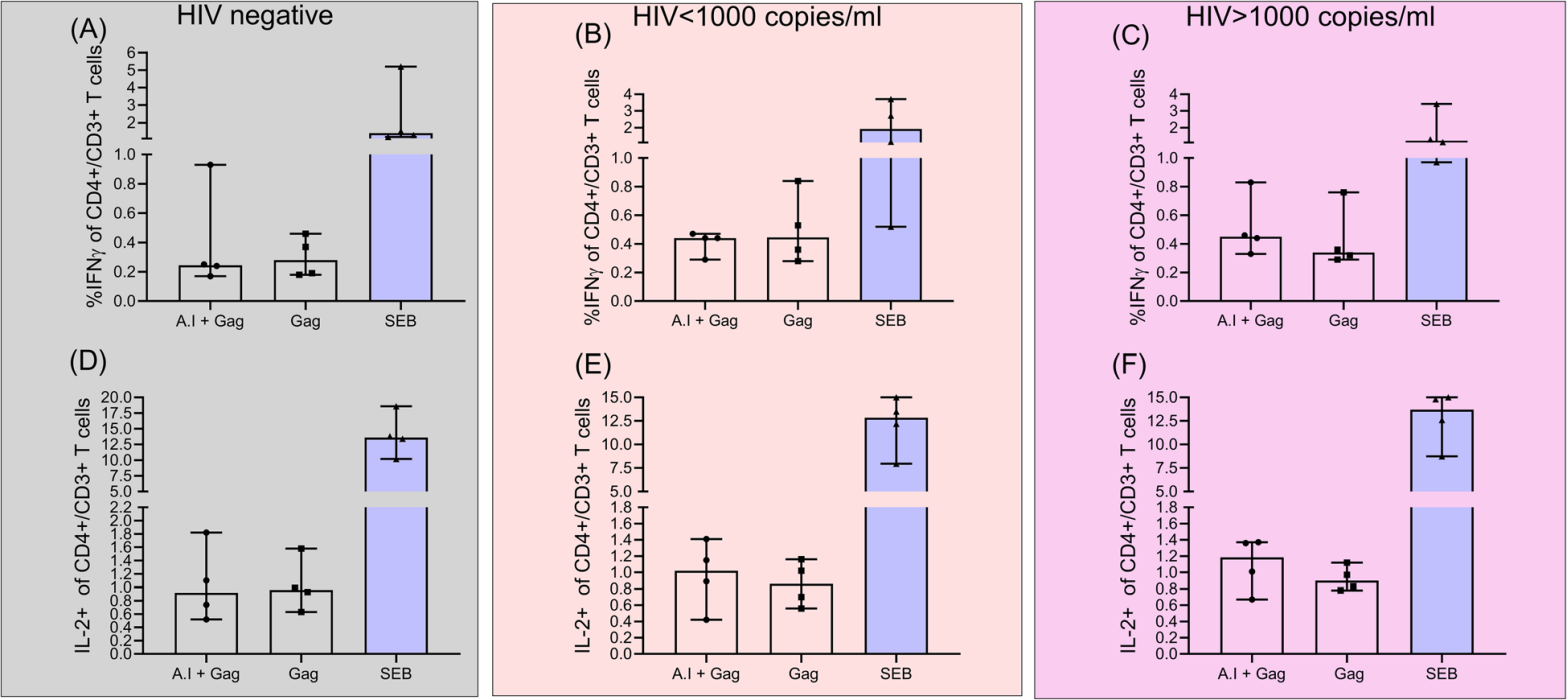
*A. indica* does not affect HIV associated cytokine responses within the groups of the different study participants. Percent levels of IFN γ+/CD4+/CD3+ T cells following stimulation with gag, *A. indica* (A.I) + gag, SEB (control) in (**a**) HIV negative (**b**) viral load < 1000 copies/ mL and (**c**) viral load > 1000 copies/ mL study groups. Levels of IL-2+/CD4+/CD3+ T cells after separate exposure to gag, *A. indica* (A.I) + gag, SEB (control) conditions in in (**d**) HIV negative (**e**) viral load < 1000 copies/ mL and (F) viral load > 1000 copies/ mL study groups. Each datapoint represents results obtained from individual donor samples. Statistical analysis was performed out using the Wilcoxon matched-pairs signed rank test

**Fig. 5:**
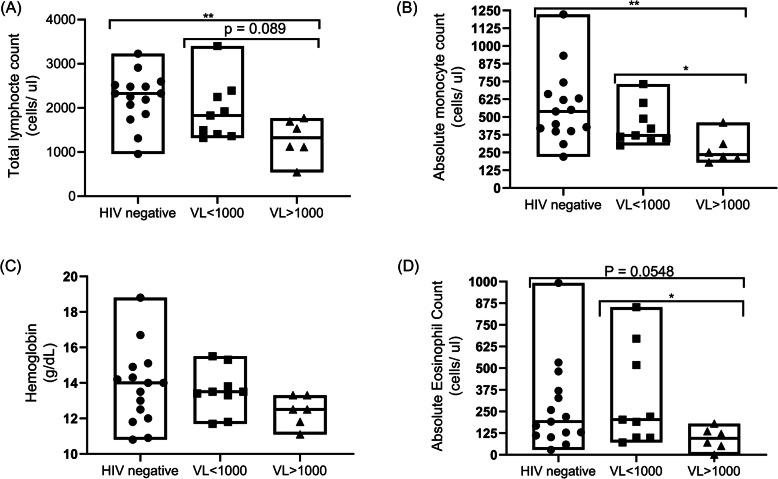
Differences in key complete blood count profiles among the study groups reveals progressive immune deficiency could account for differences in response to *A. indica*’s down-modulation of CD4+ T cell activation. Absolute counts of (**a**) Total Lymphocyte (**b**) monocytes (**c**) Haemoglobin (**d**) Eosinophils amongst HIV negative, viral load < 1000 and viral load > 1000 study groups of participants recruited for this study. Each datapoint represents results obtained from individual donor samples

Lastly, we turned our efforts towards testing whether *A.indica’s* downmodulation of CD4+ T cell activation/ exhaustion was accompanied by a loss in HIV specific cytokine (IFN-γ and IL-2) secretion. This was of interest to us since it has previously been shown that the engagement of T- cell activation markers such as CD38 influences the secretion of several pro-inflammatory cytokines such as IFN-γ, IL-6, GM-CSF, and IL-10 [46]. Regardless of HIV status and level of viremia, there was no significant difference between IFN-γ and IL-2 production when comparing the Gag and *A. indica* + Gag experimental conditions across all study groups (Fig. 4). This implies that *A. indica* extract did not affect CD4^+^ T cell IFN-γ and IL-2 responses following stimulation with Gag. However, variation in immune deficiency between the viral load < 1000 copies/ ml and viral load > 1000 copies/ ml study groups could account for  differences in downmodulation of immune activation seen following testing with *A. indica* (Fig. 5).

## Discussion

Our results support the utility of *A. indica* as a potential source of bioactive compounds for targeting HIV-1 associated immune activation. We exploited commonly used ethnobotanicals that are often alternatively utilized to manage co-infecting diseases like HIV, malaria, tuberculosis and helminths that partially contribute to hyper-immune activation and inflammation in Sub Saharan Africa [47–51]. In Uganda, *A. indica* was amongst the most frequently used herbal therapies utilized for the management of symptoms and opportunistic infections such as cough, fever diarrhoea, abdominal pain, sexually transmitted infections, liver and kidney disease during HIV-1 infection. Based on surveys from questionnaires administered to herbalists, A.*indica* decoctions prepared from leaves, roots, bulbs and were commonly administered as drinks [7]. Within the plant extract cytotoxicity profiles, the finding that increasing concentration of plant extracts led to progressive loss of cell viability suggests that at high dosages, plant extracts could levy a detrimental toll on cell survival [52, 53].

Findings from our study indicate that only the *A.indica* extract had a potent concentration-dependent inhibitory effect on CD4^+^ T cell activation and exhaustion following SEB stimulation. Similarly, A. Awah et al also showed that treating PBMCs with phytohemagglutinin (PHA) and a hydroacetone extract of *A.indica* leaf reduced CD38^+^ and CD69^+^ levels [54]. However, these observations were achieved using PBMCs from only one pooled HIV negative sample which may not account for personal variations in immune activation and functionality [55]. Besides, their findings could have been improved with a comprehensive exploration of CD4+ T cell markers of activation and exhaustion like CD38^+^HLADR^+^ co-expression, Tim3, CD69 and PD-1 which we have done here.

Finally, the immune-suppressive agent Cyclosporine was also noticed to downmodulate %CD4 + CD38 + HLA-DR+ levels following prolonged exposure to SEB. However, the application of Cyclosporine as a future therapeutic agent is limited since it has previously been reported to cause increases in HIV plasma RNA levels [56]. The inhibitory effect of *A. indica* on SEB-induced CD4+ T cell activation and exhaustion were dependent on HIV status and viremia of the study participants. *A. indica* had the most pronounced inhibitory effect on CD4^+^ T cell activation and exhaustion in PBMCs from HIV-1 negative participants. However, this effect declined but remained significant in participants with suppressed viremia. This was abrogated in participants with non-suppressed viremia with markers CD69 and PD-1 being the most affected.

PLWH who have high VL or active viral replication may have low CD4^+^ T cell counts and defective CD4^+^ T cell function as demonstrated by blunted T cell receptor (TCR) signalling, dysregulation of pro-inflammatory signalling cascades, decreased cytokine secretion and upregulated expression of inhibitory receptors [57, 58]. Thus, non-suppressed HIV infection is associated with systemic immune dysregulation as the immunopathology progresses towards AIDS which may, in part, explain our findings [59, 60]. While trying to corroborate this ideology, the major limitation that we faced was the fact the standard of patient care was switched to only viral load monitoring as opposed to performing both viral load and CD4+ T cell count testing simultaneously. As a result, we incorporated the use of Total Lymphocyte Count as a substitute for absolute CD4+ T cell counts for monitoring immune deficiency in HIV infected individuals from resource limited settings [61]. Collectively, the observation of similar total lymphocyte counts and absolute monocyte/ eosinophil counts within the virologically suppressed and healthy groups amidst progressive declines in the VL > 1000 individuals confirms the progressive leukocyte depletion and accompanying immune deficiency within the high viral load group.

The observation that ethanolic *A. indica* shows a dose-dependent inhibition of CD4+ T cell activation and exhaustion reveals a novel immune-modulatory role of this plant extract. Similarly, Nakanjako et al found that an 80 mg daily dose of the anti-inflammatory drug Atorvastatin during ART is also capable of reducing T-cell activation/exhaustion within this study population [62]. However, the massive rollout or implementation of statin therapy in concert with ART during HIV infection is limited by drug to drug interactions [63]. Despite several study groups showing that lowering or blockade of checkpoint inhibitors like PD-1 results in the rescue of T cell function, we did not observe any differences in cytokine specific responses to Gag in *A. indica* treated versus untreated conditions [64, 65].

It could be argued that *A. indica’s* reduction of CD4+ T cell activation/ exhaustion could be due to the result of selective depletion of activated CD4+ T cells. However, our results indicate that the selected concentration of this plant extract was capable of maintaining acceptable cell viabilities during the durations when we conducted our experiments. Thus, to accurately test whether the plant extracts will have any effects on CD4+ cell death on a grandiose scale, markers for apoptosis in addition to viability markers and cell death pathways will have to be included in future flow cytometry experiments [66, 67].

Collectively, these findings suggest that the ethanolic extract of *A. indica* is capable of limiting chronic CD4^+^ T cell activation and exhaustion without having any effects on the HIV specific cytokine response. Like Chloroquine that has also been reported to have the ability to reduce the levels of CD4 + CD38 + HLA-DR+ cells, these substances could be used as adjunct therapies to resolve low-level immune activation and inflammation that persists even after suppressive ART [68, 69]. It should be noted that ethanolic extracts of *A. indica* contain diverse phytochemical constituents ranging from flavonoids, tannins, saponin, steroids and alkaloids [70]. However, future research is required to identify the lead biomolecules contained in this plant extract that could be attenuating CD4^+^ T cell activation/ exhaustion and potential mechanisms of action.

Lastly, we acknowledge that our results could have some weaknesses. The small sample size used limits the power of our study findings. It should also be noted that we and others have used the in-vitro prolonged exposure of PBMCs to SEB to model the continuous exposure of T cells to bacterial products that goes on during HIV microbial translocation [23]. However, one requires an in-vivo model system like humanized mice or SIV infected non-human primates to accurately study and target the interdependence between chronic immune activation, microbial translocation and increasing antigen burden [71, 72]. Also, there was a high variability observed during SEB induced activation probably due to the elevated exposures to Cytomegalovirus (CMV) within this population. In separate studies, CMV infection has been reported to elevate SEB stimulated immune responses [73, 74].

## Conclusion

In summary, this study revealed that *A. indica* contains unknown phytochemicals that are capable of downmodulating chronic HIV associated CD4^+^ T cell activation/ exhaustion. Additional biomolecule identification before testing in other experimental model systems will be required to confirm the possible therapeutic utility of these lead compounds.

## Supplementary Information

**Additional file 1: Supplementary Figure 1**. Gating strategy used to delineate the levels of CD4+ T cell immune activation (%CD4 + CD38 + HLA-DR+ and CD4+ CD69+ T cells) and exhaustion (CD4+ Tim3+/ CD3+ T cells and CD4+ PD-1+/ CD3+ T cells) following stimulation under various conditions. Briefly, single cells were obtained from FSC-H vs FSC-A plots. Then live cells were obtained from exclusion of Amcyan live/ dead dye positive events. Lymphocytes were then excluded from FSCA versus SSC A plots and later CD4+ T cells excluded from CD4+ versus CD3+ plots. CD4 + CD38 + HLA-DR+ levels were then obtained from CD38 versus HLA-DR plots whilst CD4+/CD69+ cells were derived from CD69 vs HLA-DR plots. Lastly, PD-1+ and Tim 3+ CD4+ T cells were also obtained from PD-1/ Tim versus HLA-DR plots respectively

**Additional file 2: Supplementary Figure 2**. Gating strategy used to explore changes in cytokine (IFNα and IL-2) secretion. Briefly, single cells were excluded using FSCA- FSCH, then live cells obtained from gating out Amcyan positive events. Lymphocytes were then excluded from the FSC-A versus SSC-A plots and T cells further interrogated for CD4+ and CD8+ expression. Levels of IL-2 and IFN γ cells were then evaluated under FMO, A. indica + Gag, Gag and SEB conditions respectively

**Additional file 3: Supplementary document**. Questionnaire that was administered to consenting study participants who were enrolled within the study so as to capture history of previous and current herbal medicine use. In addition, details on alcohol and smoking habits were also obtained.

## Data Availability

Data and materials are available from authors upon request.

## Abbreviations

*A. indica*: *Azadirachta indicia* A. Juss
*M. foetida*: *Momordica foetida* Schumach
*M.oleifera*: *Moringa oleifera*
Cyclo: Cyclosporine A
SEB: Staphylococcal Enterotoxin B
PD-1: Programmed cell death protein 1
Tim 3: T-cell immunoglobulin and mucin domain-containing protein 3
IFN-γ: Interferon gamma
IL-2: Interleukin 2
TNF-α: Tumour necrosis factor alpha
FMO: Fluorescence minus one
PLWH: Persons living with HIV
VL: Viral load
CBC: Complete blood counts
ART: Antiretroviral therapy
PFA: Paraformaldehyde
HLA-DR: Human Leukocyte Antigen-DR
