# Supplementary material for: In-vitro Immunomodulatory activity of Azadirachta indica A.Juss. Ethanol: water mixture against HIV associated chronic CD4+ T-cell activation/ exhaustion
Source: BMC Complement Med Ther. 2021 Apr 9;21:114. doi: 10.1186/s12906-021-03288-0 (PMC8034071; doi:10.1186/s12906-021-03288-0)
Supplement: Supplementary file 3 — Additional file 3: Supplementary document. Questionnaire that was administered to consenting study participants who were enrolled within the study so as to capture history of previous and current herbal medicine use. In addition, details on alcohol and smoking habits were also obtained. [file 12906_2021_3288_MOESM3_ESM.docx]

**Questionnaire**

**Title: In vitro Screening of African Tropical Plant Extracts as Potential Targets of Chronic CD4 T Cell Activation in HIV-1 Infected Individuals**

Interviewee No………………………………..

Date……./…/…..

Do you use herbal/ plant products?

Yes No

In case you do, do you know the local/ common name of the plant from which it is obtained?

Yes No

If yes, kindly state the name of the plant/s

……………………………………………………………………………………………………

How frequent do you use this plant product/s?

Daily

Once in a week

Once in a month

Once in a year

Do you prepare the herbal/ plant products?

Yes No

In case you do, which plant parts do you use?

Leaves

Stems

Roots

Flowers/ fruits

Lastly, have you ever participated in the following activities?

| ***Activity*** | ***Response (Yes)*** | ***Response (No)*** |
| --- | --- | --- |
| Smoking cigarettes or any other form of tobacco use |  |  |
| Smoking marijuana |  |  |
| Drinking alcohol (local brew like: malwa and waragi, beer and wine) |  |  |
| Others specify: |  |  |

If the answer is yes to the question above, please the number of times in a week/ month that you are involved in the activity

| ***Activity*** | ***Daily*** | ***3 times in a week*** | ***Once a week*** | ***Once a month*** |
| --- | --- | --- | --- | --- |
| Smoking cigarettes or any other form of tobacco use |  |  |  |  |
| Smoking marijuana |  |  |  |  |
| Drinking alcohol (local brew like: malwa and waragi, beer and wine) |  |  |  |  |
| Others specify: |  |  |  |  |

Have you ever used herbal products on the same days that you took part in the above activities?

Yes No

***Thanks again for your participation. All responses collected will remain highly confidential and will only be used for the purposes of carrying out this study.***
